# FT-ICR Mass Spectrometry Imaging at Extreme Mass Resolving Power Using a Dynamically Harmonized ICR Cell with 1ω or 2ω Detection

**DOI:** 10.1021/acs.analchem.2c00754

**Published:** 2022-05-23

**Authors:** Mathieu Tiquet, Raphaël La Rocca, Stefan Kirnbauer, Samuele Zoratto, Daan Van Kruining, Loïc Quinton, Gauthier Eppe, Pilar Martinez-Martinez, Martina Marchetti-Deschmann, Edwin De Pauw, Johann Far

**Affiliations:** † Mass Spectrometry Laboratory, MolSys Research Unit, University of Liège, Allée de la Chimie 6-Quartier Agora, 4000 Liège, Belgium; ‡ Institute of Chemical Technologies and Analytics, TU Wien (Vienna University of Technology), Getreidemarkt 9/164, 1060 Vienna, Austria; § Austrian Cluster for Tissue Regeneration, TU Wien (Vienna University of Technology), Getreidemarkt 9/164, 1060 Vienna, Austria; ∥ Christian Doppler Laboratory for Skin Multimodal Imaging of Aging and Senescence, TU Wien (Vienna University of Technology), Getreidemarkt 9/164, 1060 Vienna, Austria; ⊥ Department of Psychiatry and Neuropsychology, School for Mental Health and Neuroscience, Maastricht University, Universiteitssingel 50, 6229ER Maastricht, the Netherlands

## Abstract

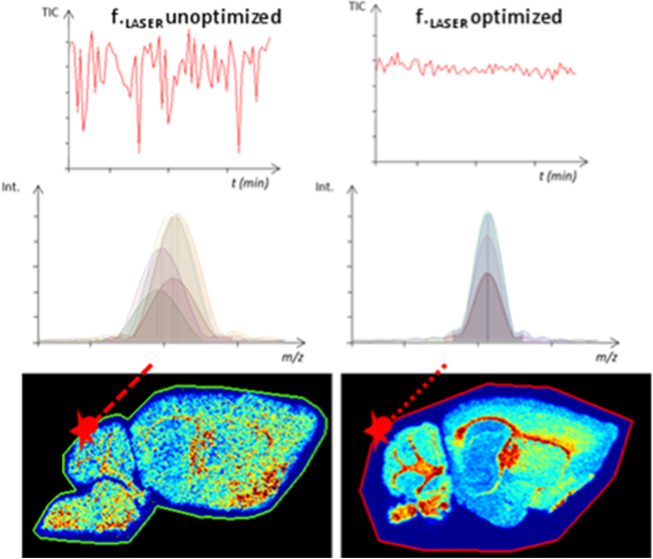

MALDI mass spectrometry imaging (MALDI MSI) is a powerful analytical method for achieving 2D localization of compounds from thin sections of typically but not exclusively biological samples. The dynamically harmonized ICR cell (ParaCell) was recently introduced to achieve extreme spectral resolution capable of providing the isotopic fine structure of ions detected in complex samples. The latest improvement in the ICR technology also includes 2ω detection, which significantly reduces the transient time while preserving the nominal mass resolving power of the ICR cell. High-resolution MS images acquired on FT-ICR instruments equipped with 7T and 9.4T superconducting magnets and the dynamically harmonized ICR cell operating at suboptimal parameters suffered severely from the pixel-to-pixel shifting of *m*/*z* peaks due to space-charge effects. The resulting profile average mass spectra have depreciated mass measurement accuracy and mass resolving power under the instrument specifications that affect the confidence level of the identified ions. Here, we propose an analytical workflow based on the monitoring of the total ion current to restrain the pixel-to-pixel *m*/*z* shift. Adjustment of the laser parameters is proposed to maintain high spectral resolution and mass accuracy measurement within the instrument specifications during MSI analyses. The optimized method has been successfully employed in replicates to perform high-quality MALDI MS images at resolving power (FWHM) above 1,000,000 in the lipid mass range across the whole image for superconducting magnets of 7T and 9.4T using 1 and 2ω detection. Our data also compare favorably with MALDI MSI experiments performed on higher-magnetic-field superconducting magnets, including the 21T MALDI FT-ICR prototype instrument of the NHMFL group at Tallahassee, Florida.

## 1 Introduction

Matrix-assisted laser desorption/ionization mass spectrometry imaging (MALDI MSI) has emerged as a label-free analytical method for monitoring the relative abundance (despite severe limitations due to suppression effects) and spatial distribution of a wide variety of analytes, especially for biological samples.^1−3^ To properly distinguish isobaric compounds^4^ inherent to the complexity of biological samples, a high resolving power at full width at half-maximum (R.P._FWHM_ > 300,000 at 400*m*/*z*) and a reliable mass measurement accuracy (MMA) are required in the absence of an upstream separation method (such as ion mobility). These performances are commonly achieved by a Fourier transform mass analyzer such as Fourier transform-ion cyclotron resonance (FT-ICR).^5^ The Bruker dual ion source ESI/MALDI FT-ICR (solariX and scimaX) is a hybrid instrument equipped with multipoles, a quadrupole, and a collision cell for precursor ion selection and fragmentation. Ions produced by electrospray (ESI) and MALDI are also accumulated in the multipole region to prepare the ion packet to be introduced into the ICR cell. Consequently, almost any combination of MALDI laser settings is compatible with any transient time for mass spectra acquisition. Recent developments introduced by Nikolaev et al.^6−8^ led to the dynamically harmonized ICR cell commercialized by Bruker in the solariX XR and scimaX XR brand FT-ICR mass spectrometers under the name ParaCell. This new cell offers the highest mass resolving power (R.P.) currently achievable for such instruments^9^ (around 1,000,000 in the lipid mass range in broadband mode), and mass measurement accuracy typically is in the sub-ppm range. These improvements drastically increase the confidence level in the determination of the empiric formula of precursor ions, especially when including the fine isotopic structure.^10^ Moreover, the introduction^11−13^ and the experimental application^12,14^ of 2ω detection drastically improved the mass R.P. and the scan duration of the ICR transient signals.

To obtain the highest quality of mass spectrometry images (MSI) in terms of mass R.P. and lateral resolution, each step of the imaging workflow has to be properly optimized. The experimental optimization of the FT-ICR-MS(I) instrument^15^ was studied by Carlos Afonso and Abdellah Tebani’s group. The sample preparation affects the ionization efficiency as well as the local diffusion of the analytes in tissue sections. Different experimental parameters were evaluated extensively in the literature including slice thickness,^16−18^ matrix and solvent selection,^19−23^ and optimization of the automatized matrix deposition.^24^ When using the recommended parameters and optimized methods intended for the previous ICR design, the “Infinity Cell”, the best performance in terms of R.P._FWHM_ and mass accuracy was far from instrument specifications due to abnormally large mass shifts. From an instrumental point of view, significant deviations in the number of injected ions between scan events heavily affect the global performance of the Paracell. Pixel-to-pixel fluctuations of the ion current during the MSI experiments cause a nonrepeatable space-charge effect between pixels in regard to the MALDI MS calibration procedure. In general, this phenomenon can be corrected using a lock-mass calibration during acquisition^25,26^ which would ideally require several homogeneously distributed analytes. These targets could be added before matrix deposition at the risk of inducing more or less severe ion-suppression effect(s) and lateral diffusion. An alternative is to use postacquisition recalibration software,^27^ which however can be time-consuming due to format conversion and computational steps depending on the size and format of the dataset.

This study reports optimized instrument conditions to mitigate the abnormal mass shifts observed during high/extreme-resolution MALDI FT-ICR MSI fitted with the ParaCell. We present here such an optimization on sample preparation and acquisition parameters to produce MS images at R.P._FWHM_ at least better than 500,000 at *m*/*z* 800 (better than 1 million at *m*/*z*: 400) in broadband mode using a solariX XR 9.4T and a scimaX 7T 2XR.

## 2 Material and Methods

### 2.1 Chemicals

Acetone and methanol (HPLC grade) were obtained from Biosolve (Valkenswaard, Netherlands). Trifluoroacetic acid (TFA, 99%), α-cyano-4-hydroxycinnamic acid (α-HCCA, purity 97%), and red phosphorus (>97%) were purchased from Sigma-Aldrich (Taufkirchen, Germany). The internal standard SPLASH LipidoMIX containing deuterated lipids from different families was purchased from Avanti Polar Lipids (Alabaster, Alabama) via Sigma-Aldrich.

### 2.2 Animal Handling

Transgenic mice were purchased from Dr. Mary Jo LaDu (University of Illinois at Chicago) and bred in-house at MHeNs at Maastricht University as described elsewhere.^28^ In short, human-APOE4 knock-in mice in which the mouse APOE gene was replaced by human APOE were crossbred with 5xFAD mice (Jackson laboratory) carrying human familial Alzheimer’s disease mutations PSEN1 and APP to obtain E4FAD mice with increased Aβ peptide production.^28,29^ Female E4FAD mice over 6 months of age were sacrificed by CO_2_ inhalation, and then brains were extracted. The mice brains were cut across the sagittal midline, immediately fresh-frozen in liquid nitrogen, and subsequently stored at −80 °C. For transportation, samples were placed on dry ice and transferred to the University of Liège to be long-term stored and conserved again at −80 °C before further handling. All procedures were approved by the Animal Welfare Committee of Maastricht University (no. AVD107002015177) and were performed according to Dutch federal regulations for animal protection.

Natural AB-type zebrafish were bred by the Groupement Interdisciplinaire de Génoprotéomique Appliquée (GIGA) at ULiège under the supervision of Pr. Marc Muller. The aquarium water was thermostated at 28 °C with a circadian cycle of 14 h of light and 10 h of darkness. One-month-old fish were first anesthetized by adding tricaine mesylate to a concentration of 0.04% and then increased to 0.16% to induce cardiac arrest. The fish were then embedded in gelatin (350 mg·mL^–1^) and stored at −80 °C for at least 24 h. All procedures were approved by the Animal Welfare Committee of the University of Liège (no. 20–2284) and were performed according to Belgian federal regulations for animal protection.

### 2.3 Tissue Sectioning

Sectioning was performed on a CryoStar NX70 (Thermo Fisher Scientific, Massachusetts) set at −20 °C. SEC35e low-profile razor blades (Thermo Fisher Scientific, Massachusetts) were employed at −15 °C during the sectioning. Mouse brain and zebrafish whole-body sagittal slices were sectioned at medium thicknesses of 14 and 8 μm to keep a good amount of material for ionization^30^ while easing the collection of seriated slices. Cryosections were thaw-mounted onto indium tin oxide (ITO)-coated conductive glass slides (Bruker Daltonics, Bremen, Germany).

### 2.4 Matrix Coating

Prior to matrix deposition, samples were dried in a vacuum desiccator for 15 min or until no visible wetness was observable. Dried samples were coated with matrix using the automatic sprayer SunCollect MALDI spotter (SunChrom, Friedrichsdorf, Germany). Matrix solution contained 5 mg.mL^–1^ α-HCCA dissolved in methanol and Milli-Q water acidified with trifluoroacetic acid (MeOH:H_2_O:TFA 9:0.99:0.01 *v:v:v*). During the spraying procedure, the nozzle was positioned to its lowest setting and its moving speeds in the *X* and *Y* axis were set at medium 10 (1540 mm·min^–1^). Matrix flow rates started from 5 μL·min^–1^ up to the fourth layer for which flow rates were increased to 10 μL·min^–1^ until the last deposition layer. The number of layers required to obtain a homogeneous coating of roughly 10 nmol·mm^–2^ of matrix was calculated for each spray deposition. The amount of matrix sprayed is confirmed by weight comparison of the ITO glass slide before and after the spray process. Later in this study, the optimized amount of deposited matrix is 5 nmol·mm^–2^.

### 2.5 MALDI Mass Spectrometry Imaging

Mass spectrometry acquisitions were performed on ESI/MALDI dual-source MALDI FT-ICRs equipped with the ParaCell (solariX XR 9.4T and scimaX 2XR 7T, Bruker Daltonics, Bremen, Germany) operating in MALDI positive mode with data point sizes of 2, 4, and 8 or 16 M for the scimaX 2XR in the 300–1200*m*/*z* mass range using the Amplitude mode. Other relevant parameters for the solariX XR 9.4T and the scimaX 7T 2XR are listed in Table 1. The shimming of the ICR cells was performed using the recommended procedure by the manufacturer based on the infusion of sodium trifluoroacetic solution in 50% acetonitrile. Before *m*/*z* calibration, the tissue to be analyzed, or a seriated tissue test section, was first probed to determine the minimum required laser power and monitor the ion current to set the laser parameters. Then, the *m*/*z* calibration of the spectrometer was performed using the odd-numbered clusters of red phosphorus spotted close to the analyzed samples.^31^ During calibration, a TIC as close as possible to the value obtained on tissue was targeted with the help of selective accumulation upper and lower cutoff set at maximum ±20% of the probed TIC on the sample. In our case, the laser powers of the solariX XR and scimaX 2XR were adjusted from 10 to 16% depending on the number of ions to be injected into the ICR cell. The laser power could be higher depending on the rate of wear of the laser. Typical high vacuum values of the ICR cells were about 2.5 × 10^–10^ mbar, and the targeted TIC with a data size of 4 M was 5 × 10^8^ cps.

**Table 1:** Sets of Parameters Used in the Original and Reoptimized Methods^a^

		solariX XR		scimaX 2XR 1 or 2ω	
parameters	(unit)	original	reoptimized	original	reoptimized
laser focus^b^	%	98	80	93	85
laser shots	(#shots)	600	[2; 10]	400	6
laser frequency	(Hz)	1000	#shots × 10	1000	60
sweep excitation power	(%)	22	[16; 18]	20	18
front & back trap plate	(V)	1.5	1.35	3	3.06
analyzer entrance	(V)	–10	–10	–10	–10
side kick	(V)	5	[6; 10]	0.2	3
side kick offset	(V)	–1.0	–1.5	–1.5	–1.5
time of flight	(ms)	1.2	1.2	1.0	0.7^c^

a The laser power was adjusted to get the lower power possible when the TIC signal was reaching 5 × 10^8^ cps. Values in brackets show a working range.

b Small and medium laser focus for solariX XR and scimaX 2XR, respectively.

c Time of flight set at 0.7 ms for the 2ω acquisition for 16 M data point only.

Automated acquisitions were performed using the software FlexImaging 5.0 (Bruker Daltonics, Bremen, Germany) with a raster of 50 μm in both (*x*,*y*) axes.

### 2.6 Data Processing

All datasets were visualized with SCiLS Lab 2016b (SCiLS, Bremen, Germany) after conversion into scilslab format using the SQlite file generated by the instrument. MALDI MSI were generated after total ion count normalization (unless specified otherwise) and automatic hot spot removal (at 99% quantile). Database bulk structure searches were performed using the LIPID MAPS Structure Database (LMSD) tool offered by LIPID MAPS Lipidomics Gateway (lipidmaps.org).^32,33^ Queries were submitted on the full database with a 5mDa mass tolerance for [M + H]^+^, [M + H-H_2_O]^+^, [M + Na]^+^, [M + K]^+^, and [M + 2Na-H]^+^ ions. The nomenclature of lipids used in this work is based on the recommended lipids classification by Fahy and co-workers.^34^ An in-house script written in R language has been used to calculate the standard deviation for MMA and R.P. for a given *m/z* window within an MSI dataset converted to imzML format by FlexImaging 5.0.

## 3 Results and Discussion

Most of the published work reporting the optimization of MALDI FT-ICR MSI methods was performed on instruments fitted with superconducting magnets of 12T and 15T or above. We propose here to visit or revisit the influence of the instrument parameters to produce MALDI images with the highest possible mass R.P. and MMA that such instruments can offer on most readily available commercial FT-ICR instruments equipped with a 7 or 9.4T magnet.

A higher magnetic field limits the space-charge effects inside the ICR cell and provides improved tolerance in regard to the number of injected ions. During MALDI MSI experiments, the amount of injected ion significantly varies due to the intrinsic heterogeneity of the biological material in terms of molecular composition and the dynamic range of the acquisition method. Consequently, instruments using lower magnetic fields could be substantially affected by impaired performance. The analytical workflow, from matrix deposition to ion optics parameters, was investigated and applied to MALDI FT-ICR instruments using 7T (scimaX 2XR) or 9.4T (solariX XR) superconducting magnets and the 1ω or 2ω detection mode, when available. Additionally, ion source parameters, only poorly explored in the literature, were explored at optimal settings to improve the quality of MALDI images at very high mass R.P.

### 3.1 Magnetic Field and Charge-Space Effects

The determination of the *m*/*z* ratios by FT-ICR is obtained by converting the rotational frequencies of the ions by Fourier Transformation, which depends on the masses and carried charges under the influence of the applied magnetic fields. The space-charge effect limits the performance of an FT-ICR due to the influence of the charge repulsion between ion packets if the ICR cell is loaded with more ions than the magnetic field can constrain. Using a superconductive magnet with higher magnetic fields would limit this influence as discussed elsewhere.^35,36^

In this paper, the main focus is on limiting the ion current fluctuation during the MSI acquisition and restricting the observed mass shift to the extent possible. This corresponds to limiting the fluctuations of the space-charge effect components at a constant magnetic field. Nonetheless, the requirement for the magnetic field to produce MALDI images at extreme mass R.P. was evaluated by comparing experimental results from 9.4T to a 7T operated in 1ω and/or 2ω detection modes.

Considering theory, the best chance to restrain the experimental mass shift in the average MS images under the specification of the FT-ICR is to prevent space-charge effects. This was investigated by experimental work and a literature survey for the different steps of the production of MALDI images, from sample preparation (matrix deposition protocols) to instrumental parameters (ion optics transmission, ICR ion optics). Furthermore, optimization works for laser adjustments are still scarce in the literature and were also investigated.

The monitored outputs of experimental parameters during measurement at very high mass R.P. (500,000 and above in the lipid mass range) were mainly the stability of the total ion current (TIC) fluctuation, the mass shift (i.e., pixel-to-pixel variation of the *m*/*z* peak apexes), and the mass R.P. (R.P. expressed as full width at half-maximum, FWHM) for the individual pixel and in the profile average mass spectrum of the image. The apparent intrascan dynamic range between major and minor peaks of lipids was also monitored and reported during the laser parameter optimization.

The effect of significant TIC fluctuations on the *m/z* shift was evaluated by monitoring the signal produced by a standard Splash LipidomiX solution of deuterated lipids spotted with α-HCCA matrix on an ITO glass slide. After the acquisition of a single MALDI MS scan involving 10 laser shots, the instrument was post-calibrated using the signals from [PC (15:0/18:1(d7)) + H]^+^*m*/*z* 753.613, [LysoPC (18:1(d7)) + H]^+^*m*/*z* 529.399, and [SM (d18:1/18:1(d9)) + H]^+^*m*/*z* 738.647. The acquired signal was observed with an R.P. above 200,000 (FWHM) at their respective *m*/*z* and MMA better than 0.5 ppm (MMA after post-calibration). When the number of injected ions was increased (i.e., using 400 laser shots), it resulted in a 10× higher total ion count injected into the ICR cell and a mass shift for all experimentally observed *m*/*z* values resulting in an MMA between 2 and 8 ppm. The larger amount of ions introduced into the cell in regard to the calibration procedure severely impaired the MMA. An abrupt modification of the TIC intentionally generated by suddenly increasing the number of laser shots was correlated with the observed mass shift. An example is provided in Figure S1 for illustration based on the signal obtained for [PC (15:0/18:1(d7)) + H]^+^.

From an MSI perspective, such TIC variations commonly appear when inhomogeneous matrix deposition creates hot spots, and/or when samples, such as tissue sections, have intrinsically heterogeneous regions in terms of molecular compositions and/or desorbed/ionized efficiencies.

### 3.2 Influence of Sample Preparation and the Amount of Sprayed Matrix on the Total Ion Current Stabilization

**Figure 1:**
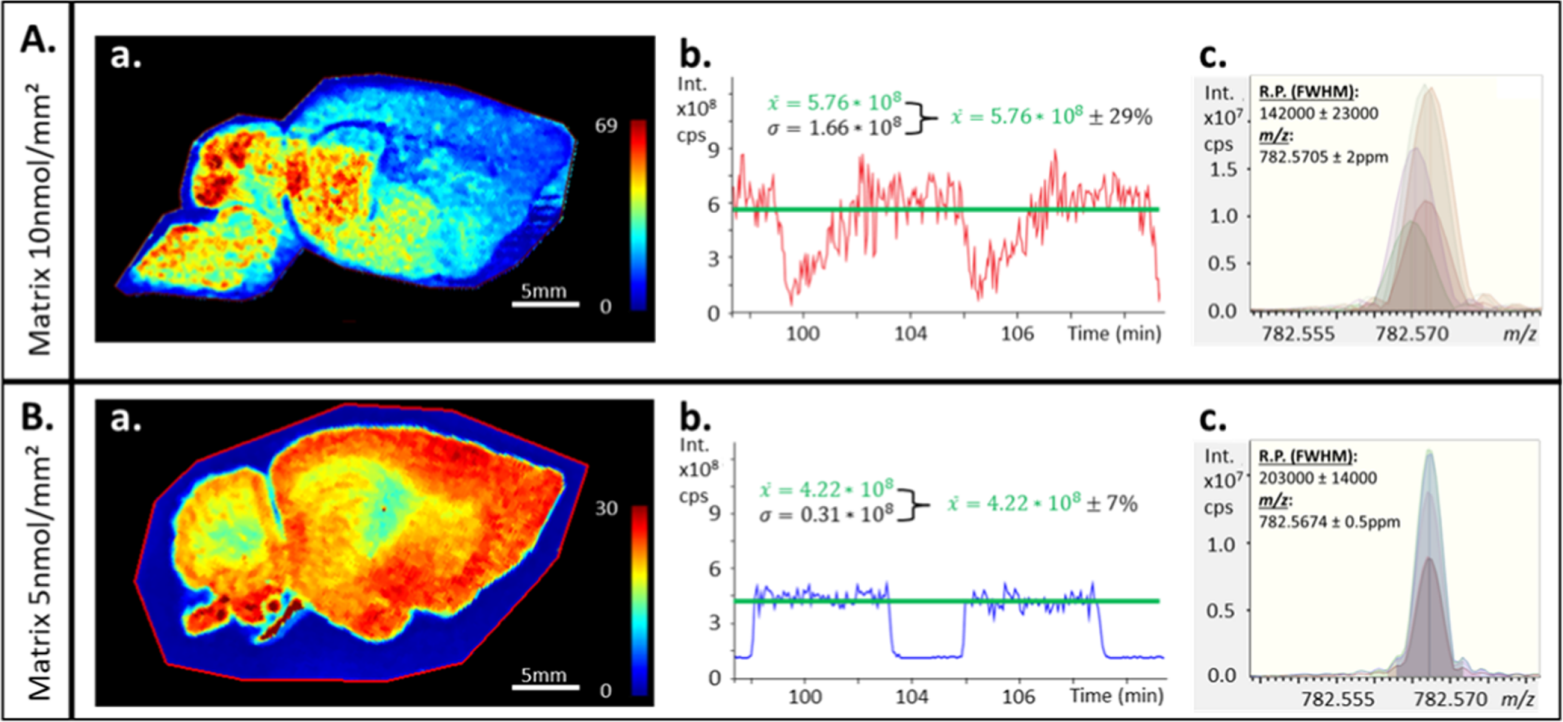
Comparison of dynamically harmonized MALDI FT-ICR MSI acquired on a solariX XR 9.4T at 4M with a manufacturer recommendation-based method (A) and a 6 laser shot-based method (B); see Section 3.2 for details. Reconstructed heat maps of the non-normalized total ion count of MS images (a). Portions of the TIC over time of the MSI acquisition and the computed mean intensities with standard deviation (b). Observable gaps on the TIC are values from pixels outside of the tissue section and were excluded to compute the standard deviation. The TIC presented in the top panel was obtained when 10 nmol·mm^–2^ α-HCCA matrix was deposited using 98% laser focus and from 5 nmol·mm^–2^ α-HCCA matrix with 80% laser focus (bottom panel). Multipixel mass spectra overlay of *m*/*z* 782.5674 shows a notable improvement in terms of mass R.P. and mass accuracy measurements during the MALDI images between the unstable (top panel) and stable (bottom panel) total ion count (c).

Avoiding the formation of hot spots due to inhomogeneous deposition of the matrix is important to produce MS images of high quality. The KPMP Consortium (Veličković et al.) and Tressler et al. improved MALDI MSI data after factorial design optimization of the deposited matrix using an automatic sprayer on mice’s kidney tissue sections.^24,37^ In the presented work, the amount of deposited MALDI matrix was investigated in terms of signal suppression for the analytes of interest when varying the number of laser shots per pixel (see Figure S2). For this purpose, serial sagittal mouse brain slices were prepared with varying amounts of sprayed α-HCCA matrix of 10 and 5 nmol per mm^2^, respectively. MS images were acquired either using several hundreds of laser shots at 1000 Hz (Figure 1A) or using 6 laser shots at 60 Hz (Figure 1B). In the latter, the laser focus of the solariX XR 9.4T Smartbeam II laser was adjusted from 98 to 80% to ablate and desorb an equivalent amount of material per pixel. This allowed the generation of similar TIC values between the MALDI MSI methods. Figure 1A shows an unstable TIC for the method employing suboptimal parameters while the new set of optimized settings showed a drastically improved TIC stability through the entire acquisition (Figure 1B). This resulted in improved alignment of the *m*/*z* peaks keeping the mass shifts below 0.5 ppm while improving effective mass R.P. in the mean spectrum to around 400,000 at *m*/*z* 400 for all detected ions while keeping the same spatial resolution (Figure 1A,B panel c). An in-house script has been used to monitor the *m*/*z* channels for every pixel and compute the distribution of their measured values (apex of the *m*/*z* peaks) and the R.P. using FWHM. Figure S3 provides a graphical representation of the mass and R.P. distribution for *m*/*z* 782.5674 ([PE 39:4 + H]^+^ or [PC 36:4 + H]^+^ (according to the LIPID MAPS database) for both methods which were drastically improved when the TIC fluctuation was restrained.

### 3.3 ICR Mass Analyzer Optimization and the Influence of Ion Optic Voltages on TIC Stability

**Figure 2:**
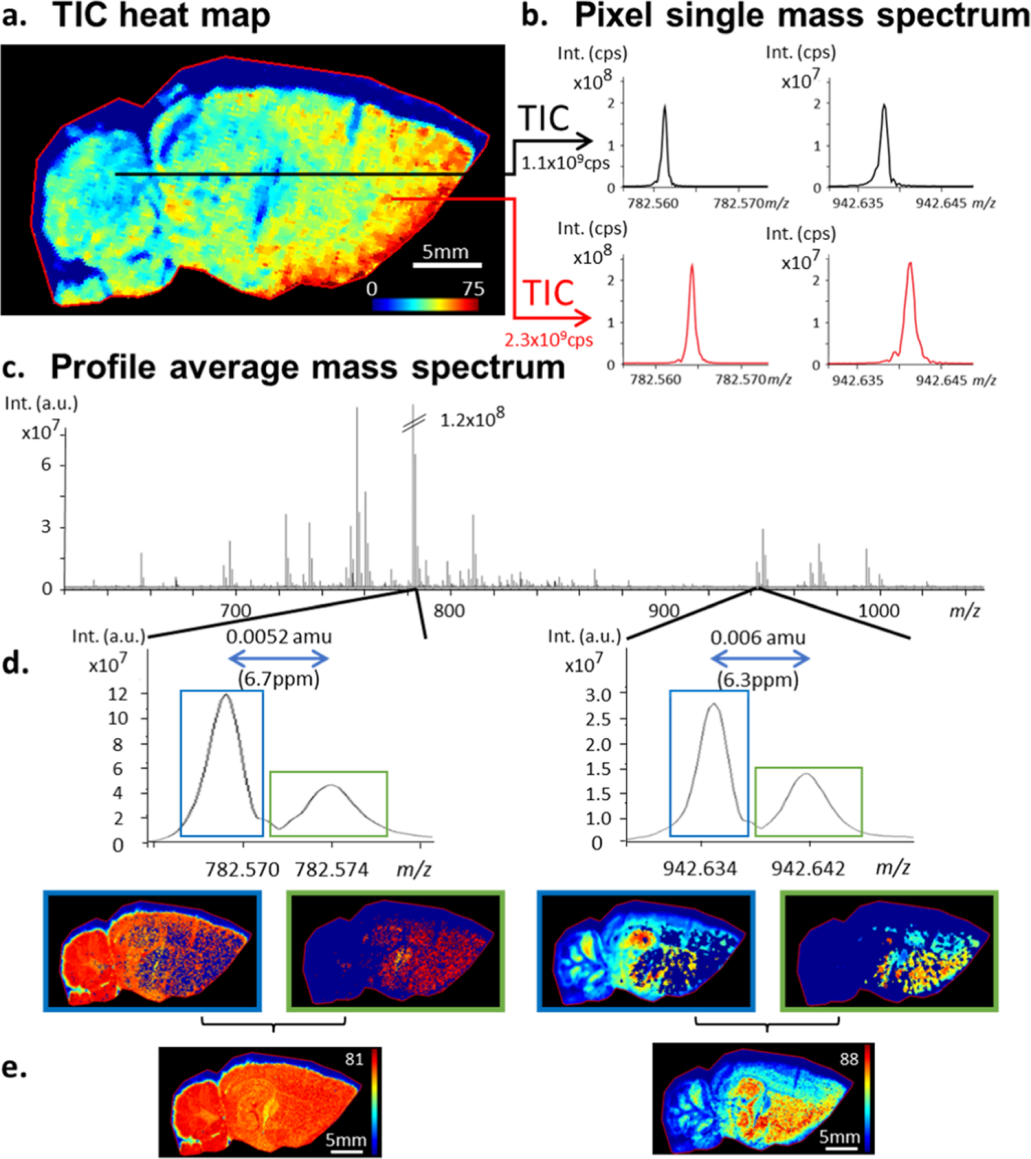
Heat map of the non-normalized TIC of a mouse brain section analyzed by high-resolution MALDI FT-ICR MSI on a solariX XR 9.4T using nonoptimized MSI method (a). Extracted mass spectra from single pixels located in regions with significant differences in total ion current (b). Average mass spectrum (mean spectrum) of the whole MALDI image (c). Zoomed profile average spectrum focused on *m*/*z* 782.57 and 942.64 showing artifacts of split peaks and their complementary distributions due to inconsistent mass measurement accuracy during acquisition (d). Obtained localizations with a window selection encompassing both *m*/*z* peaks shown in the vicinity of *m*/*z* 782.57 and 942.64, respectively (e).

First, the method employed was based on values recommended by the manufacturer for MALDI MSI. Minimal modifications were the use of the broadband mode in the 300–1200 amu mass range working at an estimated R.P. above 400.000 at *m*/*z* 800 with 4 M data point. The solariX XR 9.4T instrument was operated following a method optimized by Ferey et al.^15^ They optimized the MALDI FT-ICR MSI parameters using experimental designs from a 12T magnet instrument fitted with the Paracell. However, our MSI acquisitions performed on our 9.4T magnet suffered from severe mass shifts as shown in Figure 2. For individual pixels of the image, the experimental R.P. was slightly above the one estimated by the FT-ICR control software (FTMS control). Nevertheless, the centroids of the *m*/*z* peaks shifted from pixel-to-pixel resulting in an MMA below the specification of the instrument as observed in the profile average mass spectrum of the image. The MSI profile average spectrum showed peak broadening due to the combination of pixels mass spectra, where a significant pixel-to-pixel mass shift of the measured *m*/*z* occurred. Extreme cases were observed, where the *m*/*z* peaks were splitting by a few milli amus (i.e., several ppm), as shown in Figure 2d. The reconstructed MS images of *m*/*z* 782.57 (assumed to be [PC 34:1 + Na]^+^) and 942.64 (assumed to be [CL 36:4 + NH_4_]^+^), both selected with a mass tolerance of ±0.004, result in biased and incomplete ion distributions unless the targeted ions and their shifted counterparts *m*/*z* peak were selected together by extending the mass tolerance to ±0.01 for image reconstruction (Figure 2e). A comparison of the extracted spectra on a per-pixel basis (Figure 2a) showed that peak splitting could again be linked to the regions of interest submitted to large TIC variation despite the ion optics optimization adapted from Ferey et al. for our 9.4T instrument.

Ion optic voltages of the ICR mass analyzer were investigated as options to stabilize the TIC signal (i.e., charge-space effects) in our 9.4T FT-ICR at 4M. Thus, investigations were focused on ICR parameters, especially analyzer entrance, front and back trapping, sidekick, and excitation sweep voltages. Out of those parameters, the sidekick was the only parameter that had a slight influence on the TIC stability. The sidekick offset optimization also showed a limited effect on the mass shift, while the front and back trapping, as well as the excitation sweep, affected the peak shapes (as expected) but not the TIC stability. In contrast, the voltage applied to the analyzer entrance had an effect. Increasing the analyzer entrance voltage was followed by a gradual decrease until the absence of an MS signal. In the end, optimizing the ion optics does not significantly improve the TIC stability during MSI experiments.

### 3.4 Monitoring MALDI Processes and the Influence of the Number of Laser Shots

Laser-based ion sources in the commercially available MALDI MS instruments are typically using pulsed UV laser beams until kilohertz frequency. The main laser parameters accessible to the average user are the laser frequency, number of laser shots, laser power (usually in percent), and laser focus (how large is the area illuminated by the laser). The laser focus is usually constrained to keep the good lateral resolution of the MS images (i.e., density of pixels), and the laser power is set to the lowest value allowing the production of ions in any region of the imaged sample. Laser power also depends on how the laser is aging. Excluding the duration of the measurement of the transient ion frequencies, the duration of a scan to produce the mass spectrum of one pixel of the MS image is depending on the laser frequency and the number of laser shots set by the user. Because acquisitions with a single laser shot resulted in the absence of signals from both, the matrix and the tissue, 2 laser shots per scan were employed for the minimum number of laser shots per scan. Under this experimental condition, the laser frequency had to be lowered below 300 Hz once again due to an absence of signal above this threshold. Below the laser frequency of 300 Hz, no significant variations of the MS signal were observed (data not shown). To investigate the influence of the number of laser shots to produce an adequate number of ions resulting in high-quality mass spectra for MSI, the laser shots to laser frequency ratio was kept at 1:10, resulting in a constant laser shots step duration. This 1:10 ratio is effective in the 2–200 laser shots range because 2000 Hz is the operational limit of the SmartBeam II laser of the solariX XR and scimaX 2XR MALDI sources. By fixing the shooting duration and the time between the laser shots, we should avoid most of the kinetic relaxation influences and balance the potential biases due to the ion extraction from the MALDI plume by the ion optics. Beyond 200 laser shots, the maximal laser frequency would be used at the cost of the constant shooting step duration.

**Figure 3:**
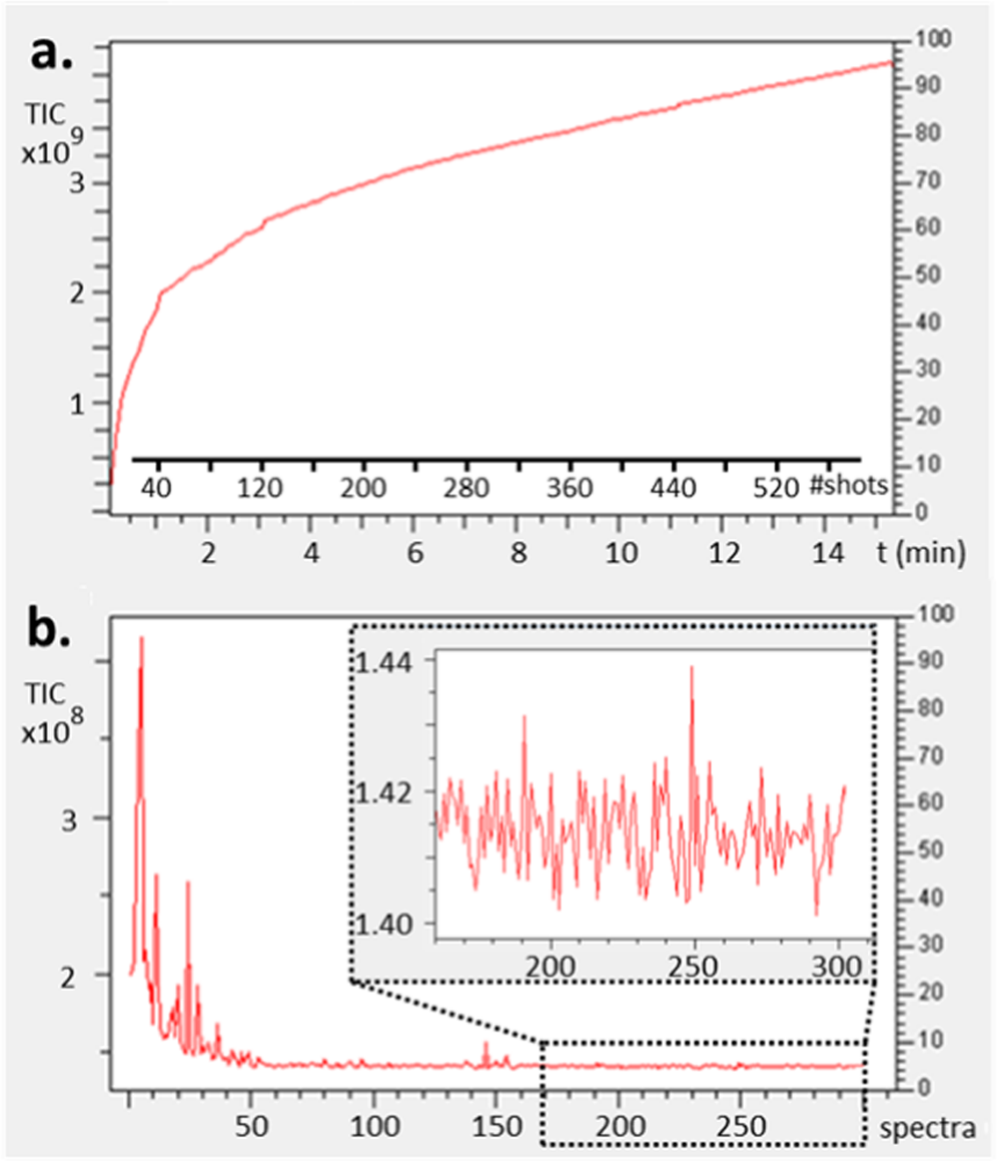
Accumulated total ion current over time (a) and total ion current per scan (b) for a 300 scans acquisition with a setting of 2 laser shots at 20 Hz per scan performed on a dynamically harmonized MALDI FT-ICR solariX XR 9.4T.

Figure 3a shows the TIC accumulation (summed TIC) of 2 laser shots at 20 Hz until the ions produced from 600 laser shots are collected, and Figure 3b shows the TIC value for each acquired individual scan. Figure 3a indicates that most of the accumulated TIC signal (more than one-half) was obtained from the first 100 laser shots and that initial laser shots produced a rather linear increase in TIC, followed by a smaller number of ions produced by subsequent laser shots. Then, a further linear increase is observed after about 120 laser shots due to the accumulation of mainly noise peaks. Figure 3b points out a notable instability of the TIC during the first 50 laser shots. The very first shots showed the highest signal abundance in the mass spectrum with a relatively high relative abundance (>10% relative intensity), which aligns with the so-called ″first-shot phenomenon″ first described by the team of Hillenkamp.^38^ The following laser shots, still ablating the same (*x*,*y*) position, only poorly contributed to good signal-to-noise ratios for interesting *m*/*z* values and the less abundant *m*/*z* peaks vanished first. These results suggest that a smaller amount of laser shots is beneficial for the detection of ions with an appropriate signal-to-noise ratio unless the targeted ions require a significantly larger amount of laser energy to be detected. Thus, using fewer laser shots, TIC fluctuations will be minimized to only a small percentage ensuring a more controlled number of ions to be injected into the ICR cell. This leads to constant space-charge effects resulting in the production of ultrahigh mass R.P. MALDI images. The contribution of the laser shots and the desorption/ionization steps of each pixel being imaged in terms of duration is typically less than 1 s from 2 to 10 laser shots when operating the laser shots to laser frequency at a 1:10 constant ratio.

### 3.5 Influence of the Number of Laser Shots on the Apparent Dynamic Range of Imaged Lipids

New sets of mice brain images were produced by MALDI MSI. The number of laser shots ranged from 10 to 600 at a fixed laser-shooting time duration (i.e., laser shots to laser frequency ratio). As expected, lowering the number of laser shots (from 600 to 10) reduced the overall signal intensities in the mass spectra (TIC) although the detected ions for both methods were comparable. The loss of *m*/*z* signals in the method using the lower amount of laser shots was mainly concerning the isotope contributions and peaks that were already close to the 3 × S/N (signal over noise) as computed by the software. Interestingly, the absolute intensities of the minor ions were almost not affected compared to the most abundant ones when using fewer laser shots and 5 nmol·mm^–2^ of deposited matrix on mice’s brain tissue sections. Table 2 reports the absolute intensity, mass accuracy, and intensity ratio between high and low abundant lipids detected in the MS images of mice’s brain tissue section when using 10 or 100 laser shots. The lipids were identified according to the LIPID MAPS database peak annotation. Ions at *m*/*z* 772.53 and 798.54 were the most intense signals observed, while *m*/*z* 770.51 and 848.56 are among the least intense ions. When comparing 10–100 laser shots, the intensities of minor ions were roughly halved while major ions’ intensities decreased by an order of magnitude. By reducing the number of laser shots per scan, the relative intensities of the most intense ions tend to decrease to a larger extent in regard to the less intense ions. Any combination of intense/less intense ion ratios leads to the same observations. Besides, the intensity ratio between ions of comparable intensities (e.g., *m*/*z* 772.53 vs 798.54) was almost unaffected by the number of laser shots per scan.

**Table 2:** Intensities and Ratios of Detected and Identified Lipids in a Mouse Brain Tissue Section Acquired with the MALDI FT-ICR MS (solariX XR 9.4T) Instrument for 10 and 100 Laser Shots

		10 shots				100 shots			
target *m*/*z*	identification	intensity (c.p.s)	mass accuracy (ppm)	ratio *m*/*z* 772.53 over *m*/*z* target	ratio *m*/*z* 798.54 over *m*/*z* target	intensity (c.p.s)	mass accuracy (ppm)	ratio *m*/*z* 772.53 over *m*/*z* target	ratio *m*/*z* 798.54 over *m*/*z* target
770.50975	[PA 36:2+K]^+^	4.8 × 10^5^	–0.09	10.6	11.3	1.3 × 10^6^	–0.12	33.1	40.0
848.55643	[PC 38:4+K]^+^	8.9 × 10^5^	–0.25	5.73	6.07	6.5 × 10^6^	–0.24	6.61	8.00
772.52519	[PC 32:0+K]^+^	5.1 × 10^6^	–0.13		1.06	4.3 × 10^7^	–0.17		1.21
798.54079	[PC 34:1+K]^+^	5.4 × 10^6^	–0.26	0.94		5.2 × 10^7^	–0.26	0.83	

The cause of the disparity in the ion intensity ratio when varying the number of laser shots was further investigated. It could indeed be related to either the ionization process itself or the efficiency of ion transmission by the ion optics and/or the MS analyzer (ICR cell). Similar experiments to determine the influence of the ionization process were conducted on a MALDI-ToF instrument (rapifleX, Bruker, Germany) despite its differences in terms of ion extraction mechanism, source vacuum, and laser compared to the solariX XR and scimaX 2XR. To be somehow comparable with the Smartbeam II, the beamscan option of the Smartbeam 3D was not used, which avoids the laser energy being swept at the surface of the sample (i.e., matrix blaster). No variation of the ion intensity ratio was observed with the MALDI-ToF, as shown in Table 3, despite we used a maximum of 1000 laser shots accumulation instead of 100, regardless of the major or minor ions considered. The ablated surface of the sample of only 25 μm^2^ with only 10 laser shots allowed the less abundant ions still to be detected, and only matrix signal intensities were strongly affected. The variation of the ion ratio observed with the MALDI FT-ICR was then related to the ion optics and/or the ICR mass analyzer. It is worth reminding that higher magnetic fields improve the dynamic range of the number of trapped ions inside the ICR in the absence of noticeable space-charge effects.

**Table 3:** Intensities and Ratio of Detected and Identified Lipids in a Mouse Brain Tissue Section Acquired with the MALDI-ToF MS (rapifleX) Instrument (External Calibration, Enhanced Cubic Regression) for 10 and 1000 Laser Shots with the Single Focus Option and Without Beamscan

		10 shots				1000 shots			
target *m*/*z*	identification	intensity (c.p.s)	mass accuracy (ppm)	ratio *m*/*z* 782.57 over *m*/*z* target	ratio *m*/*z* 798.54 over *m*/*z* target	Intensity (c.p.s)	Mass accuracy (ppm)	Ratio *m*/*z* 782.57 over *m*/*z* target	Ratio *m*/*z* 798.54 over *m*/*z* target
782.567	[PC 36:4 + H]^+^	6.8 × 10^3^	+9.71		0.79	1.8 × 10^4^	+7.1		0.81
798.541	[PC 34:1 + K]^+^	5.3 × 10^3^	+11.2	1.27		1.5 × 10^4^	+10.0	1.23	
806.567	[PS 37:0 + K]^+^	4.0 × 10^3^	–12.3	1.68	1.33	9.7 × 10^3^	–11.1	1.90	1.54
844.546	[PC 36:3 + Na]^+^	4.6 × 10^3^	+3.8	1.47	1.16	1.5 × 10^4^	6.2	1.22	0.99

### 3.6 Robustness of the MALDI MSI at Ultrahigh Mass R.P. Using the MALDI FT-ICR solariX XR 9.4T

**Figure 4:**
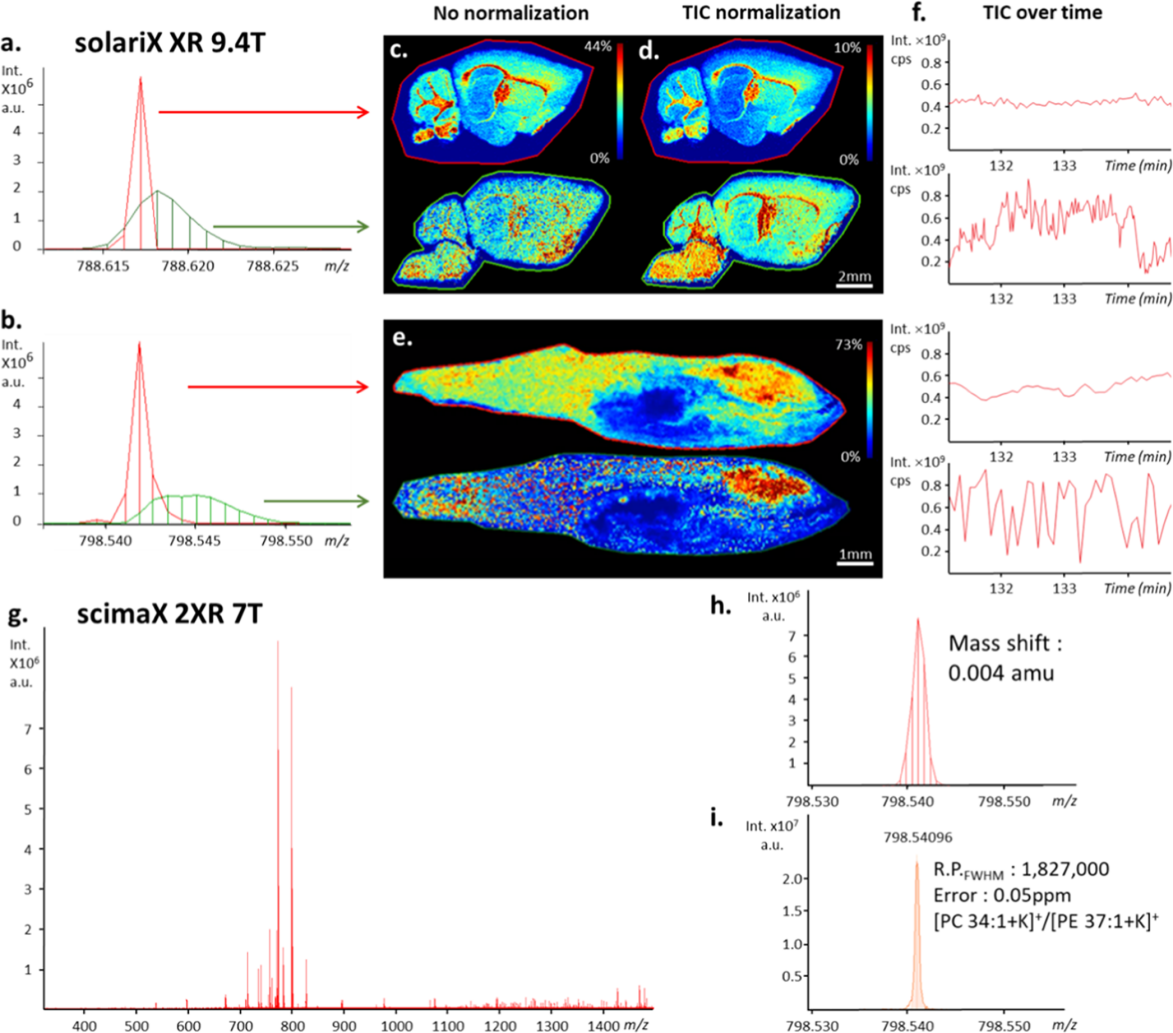
Centroided average MSI mass spectra of mouse brain (a) and zebrafish (b) using a MALDI FT-ICR (solariX XR 9.4T, fitted with ParaCell) instrument, zoomed in on *m*/*z* 788.62 and 798.54, respectively, showing peak width differences due to MMA obtained with (red) and without (green) TIC stabilization by optimization of the laser shot number (6 laser shots at 60 Hz). Reconstructed brain slices MS images without (c) and with (d) TIC normalization. Reconstructed zebrafish whole-body slices MS images with TIC normalization (e, TIC stabilization method (up) and no TIC stabilization (down), respectively). Section of the TIC over time to show the TIC stability of the presented MSI (f). Centroided average MSI mass spectrum of a mouse brain tissue section acquired on the scimaX 2XR using the 2ω detection mode with TIC stabilization (g). Zoom in to *m*/*z* 798.54 showing peak width difference due to MMA (h). Zoom in on *m*/*z* 798.54 in an extracted pixel spectrum showing the obtained R.P. FWHM and MMA (i). See text for details.

When applying the optimized method for extreme-resolution MSI with an estimated R.P. over 500,000 at *m/z* 800 (>1.000.000 at *m*/*z* 400), a much less pronounced mass shift was observed and most importantly is fully within the instrument specifications (±0.5 ppm) even for profile and centroided average MALDI image spectra. FWHM resolutions for the individual spectra are now similar to the ones observed in the profile average spectrum. The acquisition of such mass spectra qualities during an MSI experiment of rat brain samples was previously achieved using a custom prototype of a hybrid linear ion trap coupled to a 21T supra conducting magnet fitted with the Paracell by the NHMFL group at Tallahassee in Florida.^39^ In this work, comparable results were obtained in terms of MSI mass R.P. and MMA with a superconducting magnet of 9.4T using the same ICR cell (see Table S1). By comparing the average spectrum of centroided MSI data performed on seriated brain (Figure 4a) and whole-body zebrafish sections (Figure 4b), the difference in image quality is clearly evident. The new method with a controlled ion injection in the cell (TIC stabilized) resulted in narrow *m/z* peaks due to a significantly reduced mass shift. The image of the ion distribution in the tissue section is also less noisy whether or not TIC- or RMS-normalized (RMS not shown). To demonstrate the robustness of the controlled TIC method, replicates of serial brain sections (roughly 12,000 pixels) and zebrafish whole-body sections (roughly 20,000 pixels) were acquired using our optimized method (see Figure S5). In all cases, the experimental mass R.P. expected by the acquisition software was surpassed. The imaging method was tested for images with R.P._FWHM_ beyond 1,000,000 at *m*/*z* 800 for the brain region of the zebrafish sample (roughly 1500 pixels). Figure S6 shows observable isotopic fine structures for abundant ions also observable in the profile average spectrum further increasing the confidence of the identification process of these ions. Note that a slight loss in R.P. is still observed in the profile average spectrum compared to individual pixels spectra even with a contained mass shift below 0.5 ppm (i.e., in agreement with the instrument specification). At such high R.P., the contribution of a mass shift of 0.5 ppm at *m*/*z* 800 (i.e., 0.4 mamu) is still impacting negatively the R.P. In complement, a peak realignment strategy by software postprocessing coupled with our proposed MSI method was developed in our group to restore the isotopic fine structure also in the average mass spectrum of MS images having lower mass R.P..^40,41^

### 3.7 Influence of Magnetic Field Strength

FT-ICRs operating at a high magnetic field (≥12T) are typically used for petroleomics analysis by direct infusion electrospray ionization analysis of diluted raw crude oils. Direct infusion allows for a stable TIC signal and fills the ICR cell with a constant number of ions at each scan. Higher magnetic fields allow the storage of a larger number of ions and also a stable ion motion during long transient signal acquisition enabling very high mass R.P. (≥1,000,000 at *m*/*z* 400). Recently, Ge et al.^42^ demonstrated for oil samples introduced by direct infusion the capability of FT-ICR mass analyzers operating at 7T and 2ω detection to closely match the performance of a 15T instrument. MSI also takes advantage of greater magnetic fields to produce higher-quality images.^15,39^ In this work, the influence of TIC variation was investigated for an FT-ICR instrument equipped with a ParaCell but using lower magnetic field strength, i.e., 7T. The scimaX 2XR 7T instrument also provides the 2ω detection mode, which recycles the excitation plates into detection plates to improve the duty cycle (transient signal) by a factor of 2 compared to the 1ω detection mode. Mice brain images were compared for both 1ω and 2ω detection. Also, for this instrument, TIC control improved the MMA and spectral resolutions (see Figures 4f–h and S7). Interestingly, while no direct influence on ion current stability was observed when the 2ω detection mode was activated (Figure S8a), the mass shift was easier to constrain compared to 1ω mode datasets (Figure S8b). We assumed that the drastic diminution of the transient signal duration prevented peak coalescence as well as the decoherence of the ion packets inside the ICR cell.^8,43^ It is worth mentioning that the comparison of MS images performed on serial tissue sections using 1ω and 2ω detection mode showed no tangible differences in terms of co-localization of the observed ions (Figure S8b). As an illustration, an MS image acquired with the solariX 9.4T and the scimaX 2XR 7T using the 2ω detection mode at 16M data points of 1000 pixels of mouse brain tissue section showed an R.P._FWHM_ above 1,500,000 at *m*/*z* 800 and an MMA of 0.15 ppm (Figure 4f–h). The typical time to produce an image of 1000 pixels was 195 min using our solariX XR 9.4T (i.e., 1ω at 8M data point) and 205 min using the scimaX 2XR 7T operating at 2ω and 16 M data point.

Table 4 shows the dynamic range obtained for high and low abundant lipid signals detected during the MSI experiment of 2 consecutive brain sections with the scimaX 2XR using 1 or 2ω detection mode. Note that the 7T instrument still required the ICR cell to be loaded with fewer ions than the 9.4T instrument using 1ω or 2ω detection to restrict the experimental mass shift in average mass spectra. Therefore, fewer laser shots were used to produce the data in Table 4 compared to Tables 2 and 3. The ratios obtained for the scimaX 2XR using 1ω detection are somewhat similar to that obtained with the 9.4T solariX XR. The 2ω detection mode seems to be also beneficial because the dynamic range of the lipids detected in the MALDI images was less affected than the 1ω detection mode. The higher power of the magnet is still beneficial for reaching a wider intrascan dynamic range, or if the TIC cannot be efficiently stabilized, even after optimizing the laser parameters. Nevertheless, MSI acquisition at extreme mass R.P. is possible using the 7T superconducting magnet and 2ω detection.

**Table 4:** Intensities and Ratio of Detected and Identified Lipids in a Mouse Brain Tissue Section Acquired with the MALDI FT-ICR-MS (scimaX 2XR 7T) Instrument for 6 and 400 Laser Shots^a^^,^^b^

		6 laser shots				400 laser shots			
target *m*/*z*	identification	intensity (c.p.s)	mass accuracy (ppm)	ratio *m*/*z* (a) over *m*/*z* target	ratio *m*/*z* (b) over *m*/*z* target	intensity (c.p.s)	mass accuracy (ppm)	ratio *m*/*z* (a) over *m*/*z* target	ratio *m/z* (b) over *m*/*z* target
**scimaX 2XR 7T, 1ω detection mode**									
713.45181	[PA 34:1+K]+	8.0 × 10^5^	+0.40	6.63	30.0	2.7 × 10^6^	+0.61	7.04	18.5
844.52531	[PC 38:6+K]+	2.4 × 10^5^	+0.53	22.1	100	not detected	+0.58	N.C.	N.C.
772.52519	[PC 32:0+K]+	5.3 × 10^6^	+0.37		4.53	1.9 × 10^7^	+0.56		2.63
798.54079	[PC 34:1+K]+	2.4 × 10^7^	+0.26	0.22		5.0 × 10^7^	+0.44	0.38	
**scimaX 2XR 7T, 2ω detection mode**									
713.45181	[PA 34:1+K]+	4.3 × 10^6^	+0.23	3.72	4.88	4.9 × 10^6^	+0.40	4.29	10.4
844.52531	[PC 38:6+K]+	3.9 × 10^6^	+0.17	4.10	5.38	4.3 × 10^6^	+0.33	3.13	7.61
772.52519	[PC 32:0+K]+	1.6 × 10^7^	+0.22		1.31	2.1 × 10^7^	+0.37		2.43
798.54079	[PC 34:1+K]+	2.1 × 10^7^	+0.26	0.76		6.7 × 10^7^	+0.31	0.41	

a *m*/*z* (a) corresponds to 772.53, and *m*/*z* (b) corresponds to 798.54.

b N.C. Not Computed.

### 3.8 Improvement of Peak Annotation

Finally, database queries for mass lists obtained with the optimized method showed an improvement in terms of peak annotations: fewer false positives and negatives were observed due to the improved R.P. and MMA. Figure S9 shows some examples of database results as histograms of the matching counts at a given mass accuracy (in ppm) to detect readily any oddities in the dataset. When the TIC was not stabilized (not optimized method), most of the identifications had mass accuracy around −1.5 ppm. These values were not consistent with the specification of a properly calibrated FT-ICR instrument and they do not fit with the requirement for proper annotation of lipids from the LIPID MAPS database. In contrast, the MALDI image acquired with the optimized method and stabilized TIC led to a larger number of identifications, with scores around +0.4 ppm that are well within the nominal performance for the instrument. The number of total matches is drastically improved due to fewer false-negative identifications and, similarly, potentially fewer false-positive results. Of course, the addition of the isotopic fine structure further improved the confidence level of the identified lipids.

## 4 Conclusions

In this work, we successfully limited the space-charge effects and limited the resulting mass shift to improve the mass accuracy for MALDI MS images of mouse brains and Zebrafish tissue sections by introducing a controlled TIC injection method in the ICR cell. The method was successfully applied on the solariX XR 9.4T and the scimaX 2XR 7T, two commercially available dual-source ESI/MALDI instruments fitted with the Paracell. Under optimal instrumental settings, this was achieved primarily by optimizing laser parameters and the concentration of deposited/sprayed matrix. MSI with a resolving mass power beyond 1,000,000 at *m*/*z* 800 was successfully achieved within around 200 min for 1000 imaged pixels (transient duration of 11.7 s at 8M data points in an operated mass range between *m*/*z* 300 and 1200 using the common Amplitude mode for the solariX XR 9.4T, and a transient of 12.3 s for the scimaX 2XR 7T in 2ω detection mode at 16 M data points) with no mass shift beyond 1 ppm (typical mass shift <0.5 ppm), which correlates to approximately 0.5 mamu in the lipid mass range. The resulting images appeared sharper and showed improved contrast at constant lateral resolution and matrix deposition method. Extreme-resolution MS images obtained with relatively limited power of magnetic fields (<12T) require a stabilized TIC throughout the acquisition to retain the instrument specifications. The intrascan dynamic range obtained during this work using the commercially available 9.4T and 7T instruments seemed to be around 100, while Bowman et al.^39^ reported a dynamic range of around 500 using a custom 21T MALDI FT-ICR instrument. Using 2ω detection on higher-magnetic-field instruments will speed up the scan time by a factor of 2, allowing more samples to be measured at constant mass R.P. in the same time frame. Peak annotations using the LIPID MAPS database correspond to identification scores better than 0.4 ppm, limiting misidentification of lipids, especially for measurement generating isotopic fine structures. Revisiting the laser parameters improved method reproducibility from pixel to pixel and also from sample to sample, which improved the robustness of our method by successfully performing similar MALDI images of consecutive tissue sections in replicates.

It was found that the entrance voltage to the analyzer affects the number of ions introduced into the ICR cell in an interesting way that could potentially be used to limit the overflow of ions to be injected into the ICR cell, acting as an ion injection control device. The idea would be to limit ion current fluctuations in real time for samples with high-concentration heterogeneity of target compounds. This would require further investigation as it is currently considered a double-edged sword, as the signal can be easily lost if this voltage value is not properly set.

## Supporting Information Available

Trace of the TIC obtained from a deuterated lipid using two different laser parameters; comparison of the profile average MSI spectra at different laser settings; distribution of an ion mass-to-charge ratio measurement and FWHM resolution distributions at different laser settings; co-localization of one of the lipid during MSI acquisitions on same tissue but different laser settings; additional MSI replicate on brain tissues section and Zebrafish tissue sections; lipid identification using the isotope fine structure of one of the ions detected during a MALDI mass spectrometry image at mass resolving power better than 1 million; traces of the TIC and influence of TIC stability for a scimaX 7T operating in 1ω detection mode; performance of the scimaX 7T using 1 and 2ω detection mode before and after optimization; distribution of the matching exact masses with the LIPID MAPS structure database before and after method optimization; MSI performances of FT-ICR fitted with dynamically harmonized cells and using different superconducting magnets of 7T, 9.4T, and 21T instruments with 1 or 2ω detection mode; and plot representing the constant laser shot-to-laser frequency ratio with the graphical representation of the limitation of the operational laser settings (PDF)
