# Supplementary material for: FT-ICR Mass Spectrometry Imaging at Extreme Mass Resolving Power Using a Dynamically Harmonized ICR Cell with 1ω or 2ω Detection
Source: Anal Chem. 2022 May 23;94(26):9316–26. doi: 10.1021/acs.analchem.2c00754 (PMC9260710; doi:10.1021/acs.analchem.2c00754)
Supplement: Supplementary file 1 — ac2c00754_si_001.pdf [file ac2c00754_si_001.pdf]

Supporting information

FTICR Mass spectrometry imaging at extreme mass resolving power using a dynamically harmonized ICR cell with 1ω or 2ω detection

Mathieu Tiquet[1], Raphaël La Rocca[1], Stefan Kirnbauer[2,3], Samuele Zoratto[2,3,4], Daan van Kruining[5], Loïc Quinton[1], Gauthier Eppe[1], Pilar Martinez-Martinez[5], Martina Marchetti-Deschmann[2,3,4], Edwin De Pauw[1], Johann Far[1,*]

[1] Mass Spectrometry Laboratory, MolSys Research Unit, University of Liège; Allée de la Chimie 6 - Quartier Agora, 4000 Liège, Belgium.
[2] Institute of Chemical Technologies and Analytics, TU Wien (Vienna University of Technology), Getreidemarkt 9/164, 1060 Vienna, Austria.
[3] Austrian Cluster for Tissue Regeneration, TU Wien (Vienna University of Technology), Getreidemarkt 9/164, 1060 Vienna, Austria.
[4] Christian Doppler Laboratory for Skin Multimodal Imaging of Aging and Senescence, TU Wien (Vienna University of Technology), Getreidemarkt 9/164, 1060 Vienna, Austria.
[5] Department of Psychiatry and Neuropsychology, School for Mental Health and Neuroscience, Maastricht University, Universiteitssingel 50, 6229ER Maastricht, the Netherlands.
* Corresponding author: johann.far@uliege.be

* Corresponding author: johann.far@uliege.be

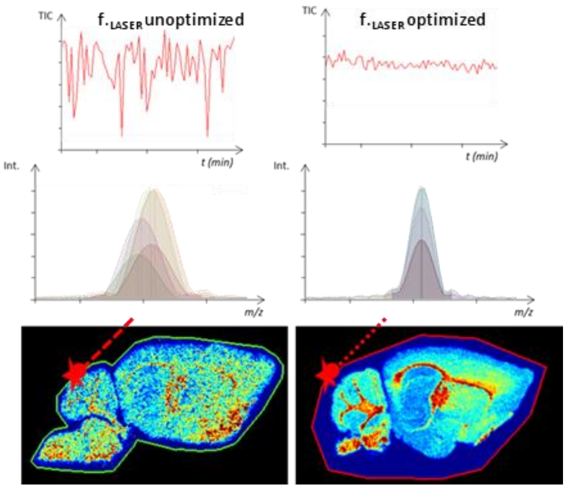

Table of Content

SUPPLEMENTARY FIGURES 4

Figure S1. TIC vs time of a MALDI FT-ICR acquisition where an increase of ions injected in the dynamically harmonized ICR cell is artificially induced at approximatively 3min (a). Observable mass shift for m/z 753.61 between the mass spectra recorded before (blue, 10 laser shots per scan) and after (red, 400 laser shots per scan) the TIC increase shown in part (b). 4

Figure S2. Profile average MSI mass spectra acquired on a MALDI FT-ICR instrument (solariX XR 9.4T, fitted with ParaCell) from mouse brain sections highlighting the reduced presence of matrix ions when the amount of matrix sprayed is reduced from 10 (a) to 5 (b) nmol.mm^{-2} of HCCA while laser parameters are switched from 400 shots with the minimum laser focus (a) to 6 shots with small laser focus setting (b)................ 5

Figure S3. Distribution of mass to charge ratio values (a) and FWHM resolutions (b) found in the window 782.570 + 10mamu for the original acquisition method, i.e. 400 laser shots and 10nmol.mm^{-2} of sprayed matrix (left) and the optimized method, i.e. 6 laser shots and 5nmol.mm^{-2} of sprayed matrix (right) throughout the entire MSI acquisition of mouse brain tissue sections.. 6

Figure S4. Localization of *m/z* 772.5338 in a pair intertwined MALDI FT-ICR MS images on a solariX 9.4T acquired with the manufacturer recommendations-based method (green) and lower amount of laser shots-based method (red) performed on the same sagittal slice with an offset of 50µm on the x-axis for the second acquisition (a). Close up on the corresponding peak in the mean spectrum of both images are provided (b) in addition to the superposition of the Total Ion Current through MSI acquisitions (c). 7

Figure S5. Replicates of two different sets of serial brain sections (roughly 12,000 pixels) and a triplicate of serial sections of whole-body zebrafish (roughly 20,000 pixels) acquired on a solariX MALDI FT-ICR MS 9.4T using the optimized method demonstrating its robustness. 8

Figure S6. Representation of the average mass spectrum of an extreme resolution broadband MSI acquisition performed between *m/z* 400 to 1200 on a zebrafish brain accounting for 2000 pixels (upper panel). Zoom into the different isotopes of the most abundant ion where the isotopic fine structure is observed (middle panel). Image of the ion distribution of *m/z* 798.5417 in the brain of a zebrafish (bottom left panel). Results from a database search in LIPID MAPS showing all matches with a 5 mDa tolerance (bottom right panel)... 9

Figure S7. Total Ion Current over time of mass spectrometry image acquisitions on a scimaX 2XR 7T in 1ω mode without controlling the ion current (red) and while controlling the ion current (green) (a). MS images mean spectra close up on the distribution of 798.54 *m/z* showing the limited mass shift obtained when using a method limiting TIC fluctuations (right) compared to a non-TIC controlling method (left) (b). 10

Figure S8. Total Ion Current over time for MSI acquisitions on a scimaX 2XR 7T in 1ω mode (orange) and 2ω mode (blue) (a). Centroided average mass spectra and distributions of *m/z* 772.53, 798.54 and 826.57 with a close-up on *m/z* 798.54 to compare the mass shift obtained with 1ω mode (right) and 2ω mode (left) (b). 11

Figure S9. Representations of bulk searches using LIPID MAPS structure database (LMSD) submitting peak lists from MSI experiments. The number of matches versus

their respective mass accuracy in ppm is presented. MALDI dynamically harmonized FT-ICR MSI datasets shown were acquired with unstable Total Ion Current (a) and stabilized Total Ion Current (b). 12

SUPPLEMENTARY TABLE 13

Table S1. Figures of merit of MSI images produced on 7T, 9.4T, and 21T MALDI FT-ICR instruments fitted with the dynamically harmonized cell. 13

SUPPLEMENTARY GRAPH 14

Graph S1. Logarithmic scale of the number of laser shots per second, i.e. frequency, versus the number of laser shots per MALDI ionization step, i.e. laser shots per scan. The laser frequency upper (2000Hz) and lower (20Hz) limits of the laser are indicated by blue lines and the effective ratio used to keep the laser-shooting time duration constant is shown by the black line. The slope corresponds to a ratio laser shots to laser frequency of 1:10........ 14

SUPPLEMENTARY FIGURES

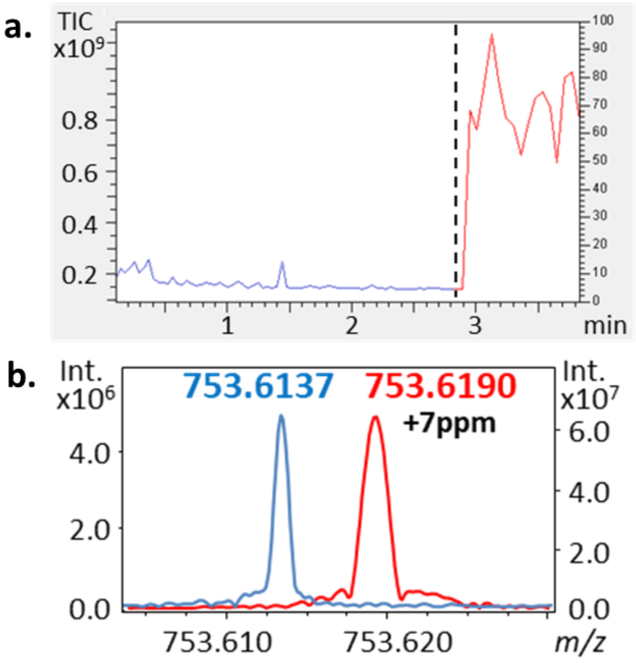

Figure S1. TIC vs time of a MALDI FT-ICR acquisition where an increase of ions injected in the dynamically harmonized ICR cell is artificially induced at approximatively 3min (a). Observable mass shift for m/z 753.61 between the mass spectra recorded before (blue, 10 laser shots per scan) and after (red, 400 laser shots per scan) the TIC increase shown in part (b).

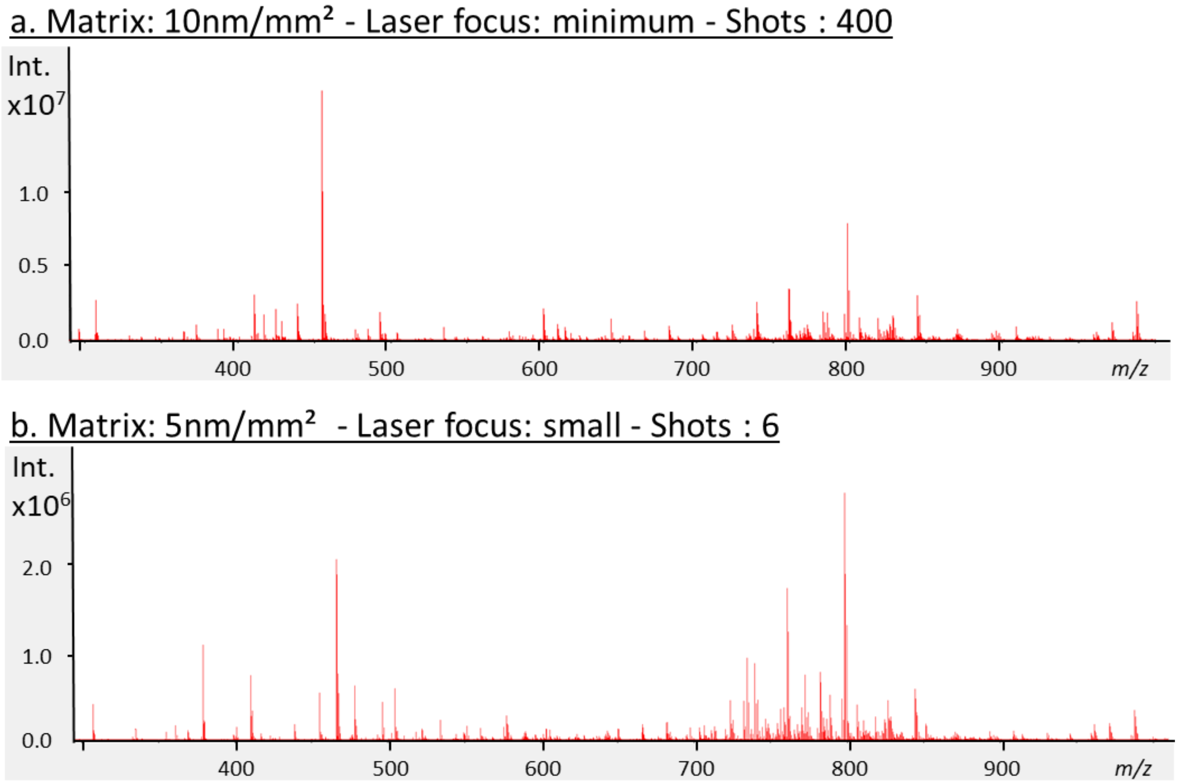

Figure S2. Profile average MSI mass spectra acquired on a MALDI FT-ICR instrument (solariX XR 9.4T, fitted with ParaCell) from mouse brain sections highlighting the reduced presence of matrix ions when the amount of matrix sprayed is reduced from 10 (a) to 5 (b) nmol.mm^{-2} of HCCA while laser parameters are switched from 400 shots with the minimum laser focus (a) to 6 shots with small laser focus setting (b).

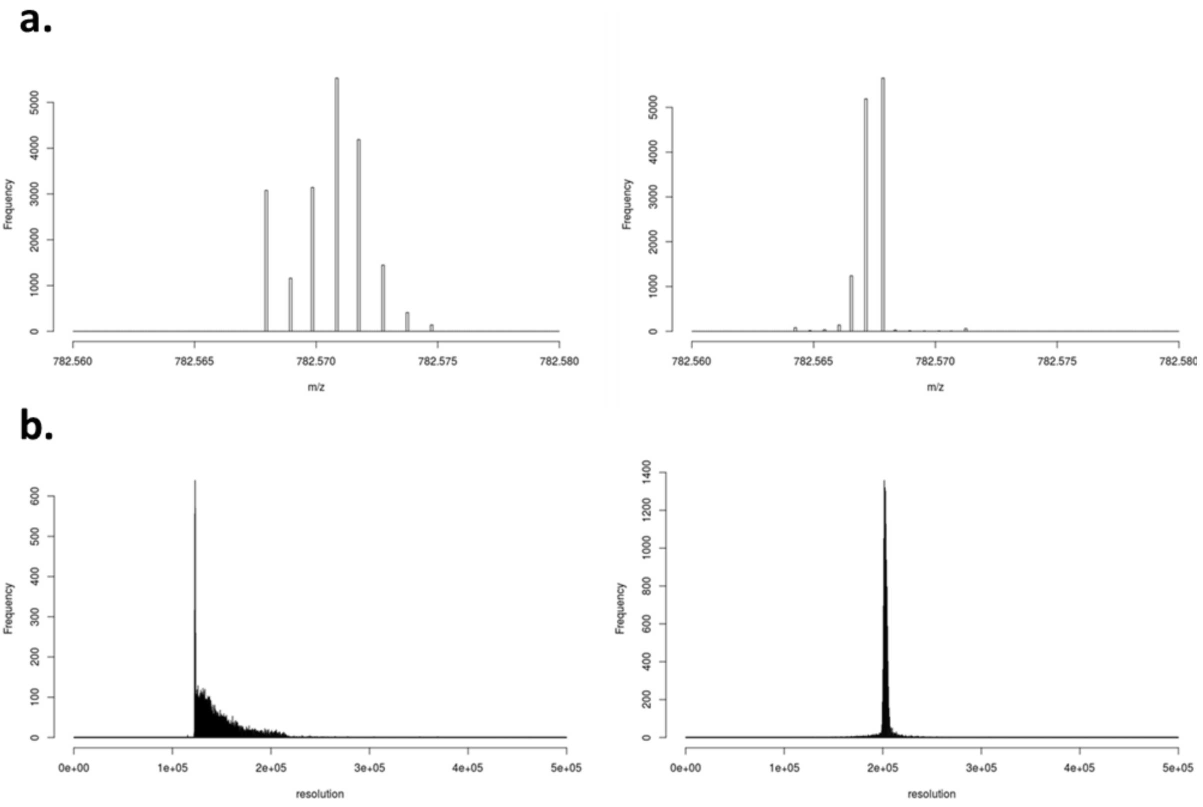

Figure S3. Distribution of mass to charge ratio values (a) and FWHM resolutions (b) found in the window 782.570 + 10mamu for the original acquisition method, i.e. 400 laser shots and 10nmol.mm^{-2} of sprayed matrix (left) and the optimized method, i.e. 6 laser shots and 5nmol.mm^{-2} of sprayed matrix (right) throughout the entire MSI acquisition of mouse brain tissue sections.

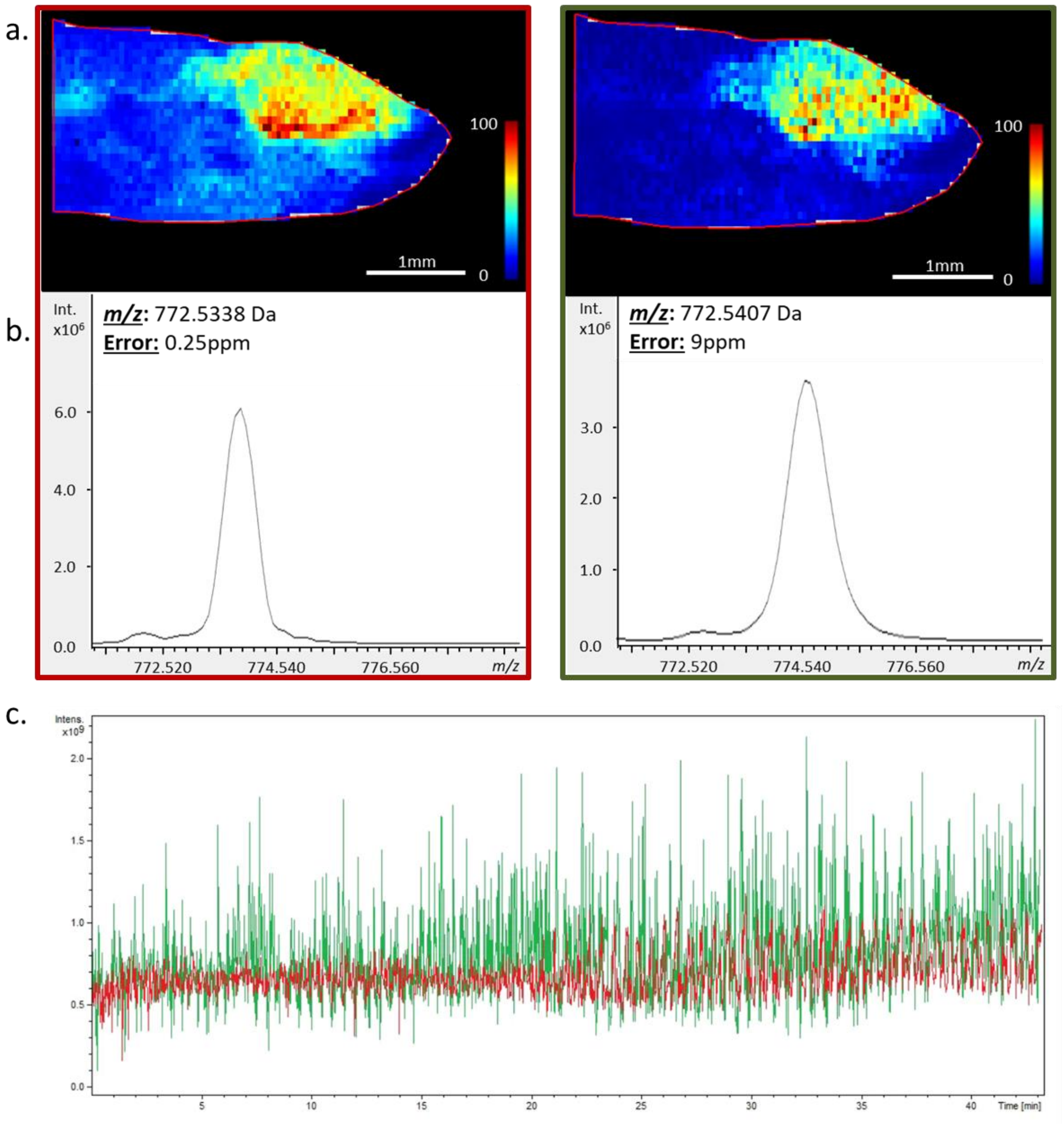

Figure S4. Localization of *m/z* 772.5338 in a pair intertwined MALDI FT-ICR MS images on a solariX 9.4T acquired with the manufacturer recommendations-based method (green) and lower amount of laser shots-based method (red) performed on the same sagittal slice with an offset of 50µm on the x-axis for the second acquisition (a). Close up on the corresponding peak in the mean spectrum of both images are provided (b) in addition to the superposition of the Total Ion Current through MSI acquisitions (c).

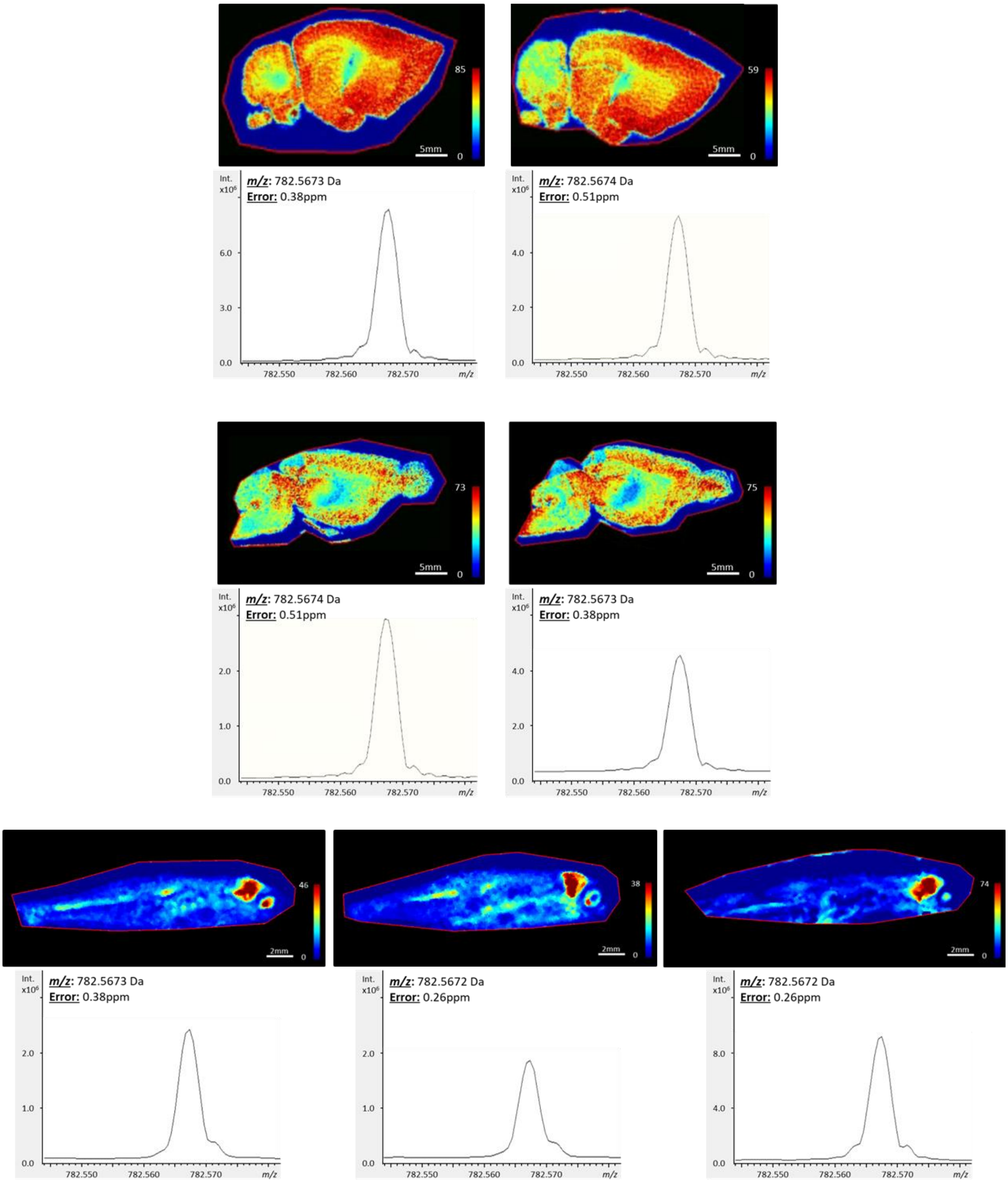

Figure S5. Replicates of two different sets of serial brain sections (roughly 12,000 pixels) and a triplicate of serial sections of whole-body zebrafish (roughly 20,000 pixels) acquired on a solariX MALDI FT-ICR MS 9.4T using the optimized method demonstrating its robustness.

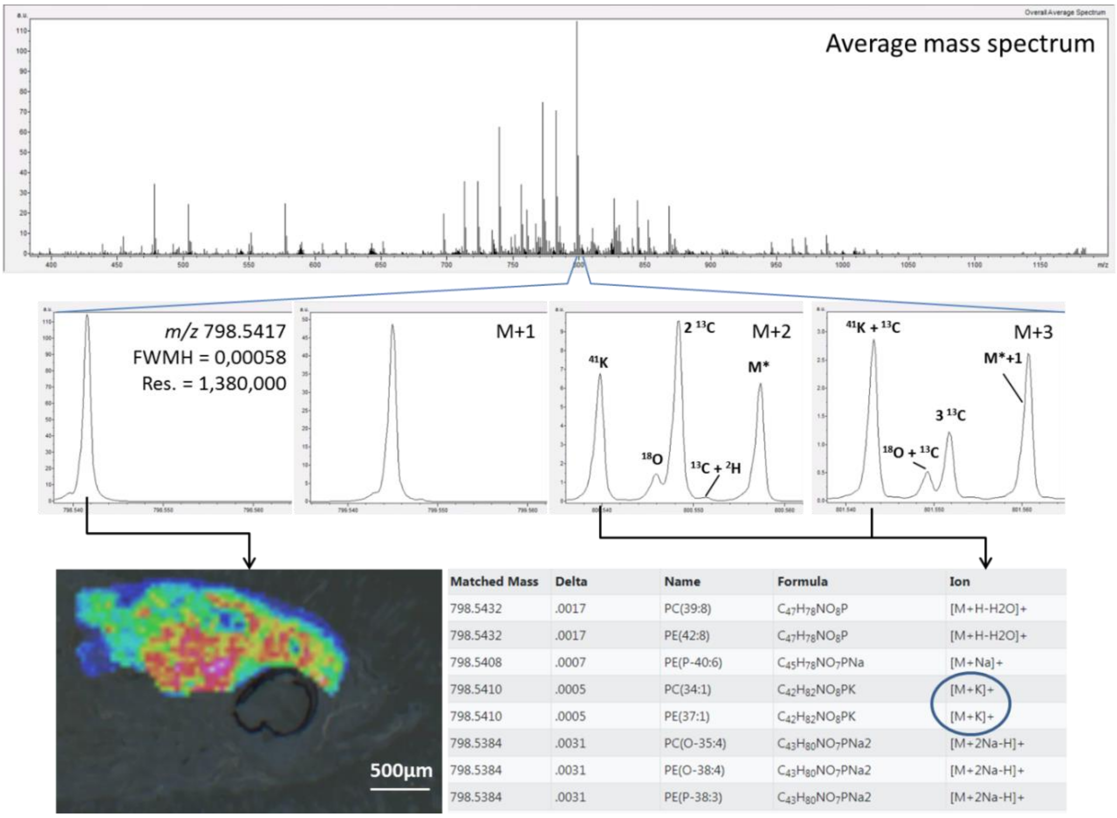

Matched Mass	Delta	Name	Formula	Ion
798.5432	.0017	PC(39:8)	$C_{47}H_{78}NO_8P$	[M+H-H2O]+
798.5432	.0017	PE(42:8)	$C_{47}H_{78}NO_8P$	[M+H-H2O]+
798.5408	.0007	PE(P-40:6)	$C_{45}H_{78}NO_7PNa$	[M+Na]+
798.5410	.0005	PC(34:1)	$C_{42}H_{82}NO_8PK$	[M+K]+
798.5410	.0005	PE(37:1)	$C_{42}H_{82}NO_8PK$	[M+K]+
798.5384	.0031	PC(O-35:4)	$C_{43}H_{80}NO_7PNa2$	[M+2Na-H]+
798.5384	.0031	PE(O-38:4)	$C_{43}H_{80}NO_7PNa2$	[M+2Na-H]+
798.5384	.0031	PE(P-38:3)	$C_{43}H_{80}NO_7PNa2$	[M+2Na-H]+

Figure S6. Representation of the average mass spectrum of an extreme resolution broadband MSI acquisition performed between *m/z* 400 to 1200 on a zebrafish brain accounting for 2000 pixels (upper panel). Zoom into the different isotopes of the most abundant ion where the isotopic fine structure is observed (middle panel). Image of the ion distribution of *m/z* 798.5417 in the brain of a zebrafish (bottom left panel). Results from a database search in LIPID MAPS showing all matches with a 5 mDa tolerance (bottom right panel).

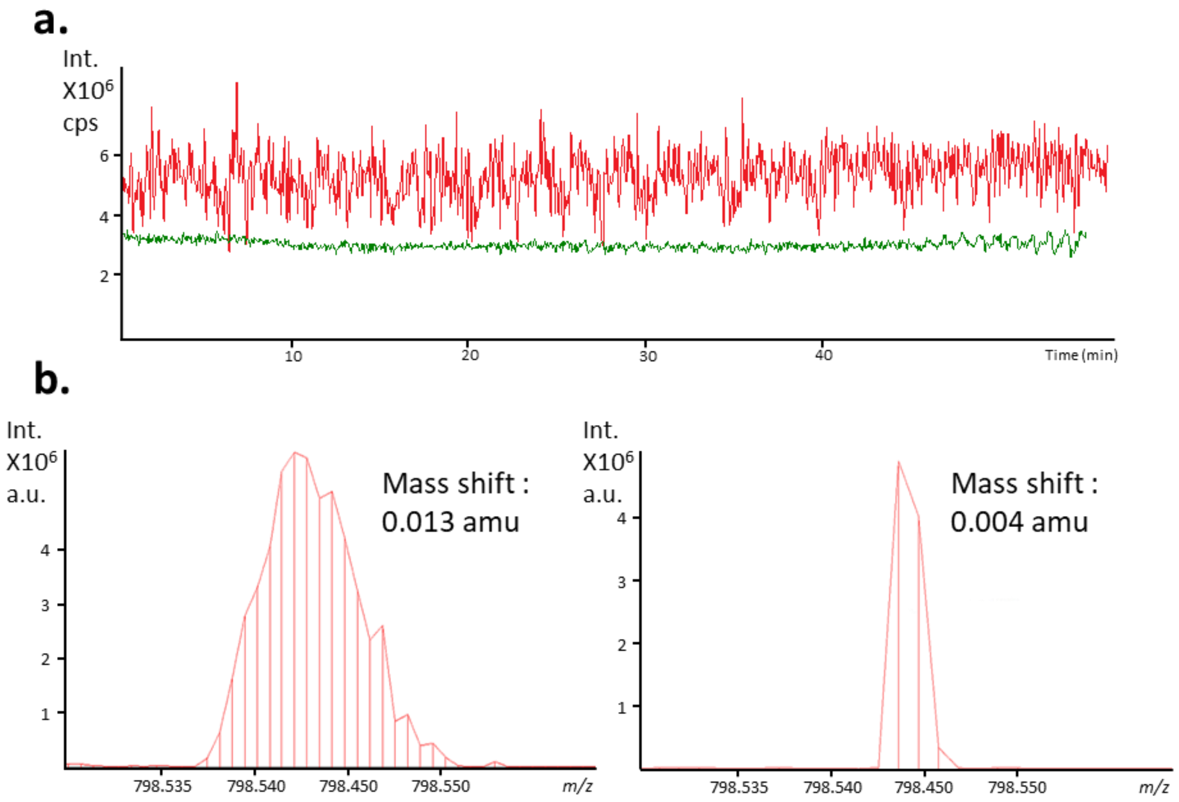

Figure S7. Total Ion Current over time of mass spectrometry image acquisitions on a scimaX 2XR 7T in 1ω mode without controlling the ion current (red) and while controlling the ion current (green) (a). MS images mean spectra close up on the distribution of 798.54 *m/z* showing the limited mass shift obtained when using a method limiting TIC fluctuations (right) compared to a non-TIC controlling method (left) (b).

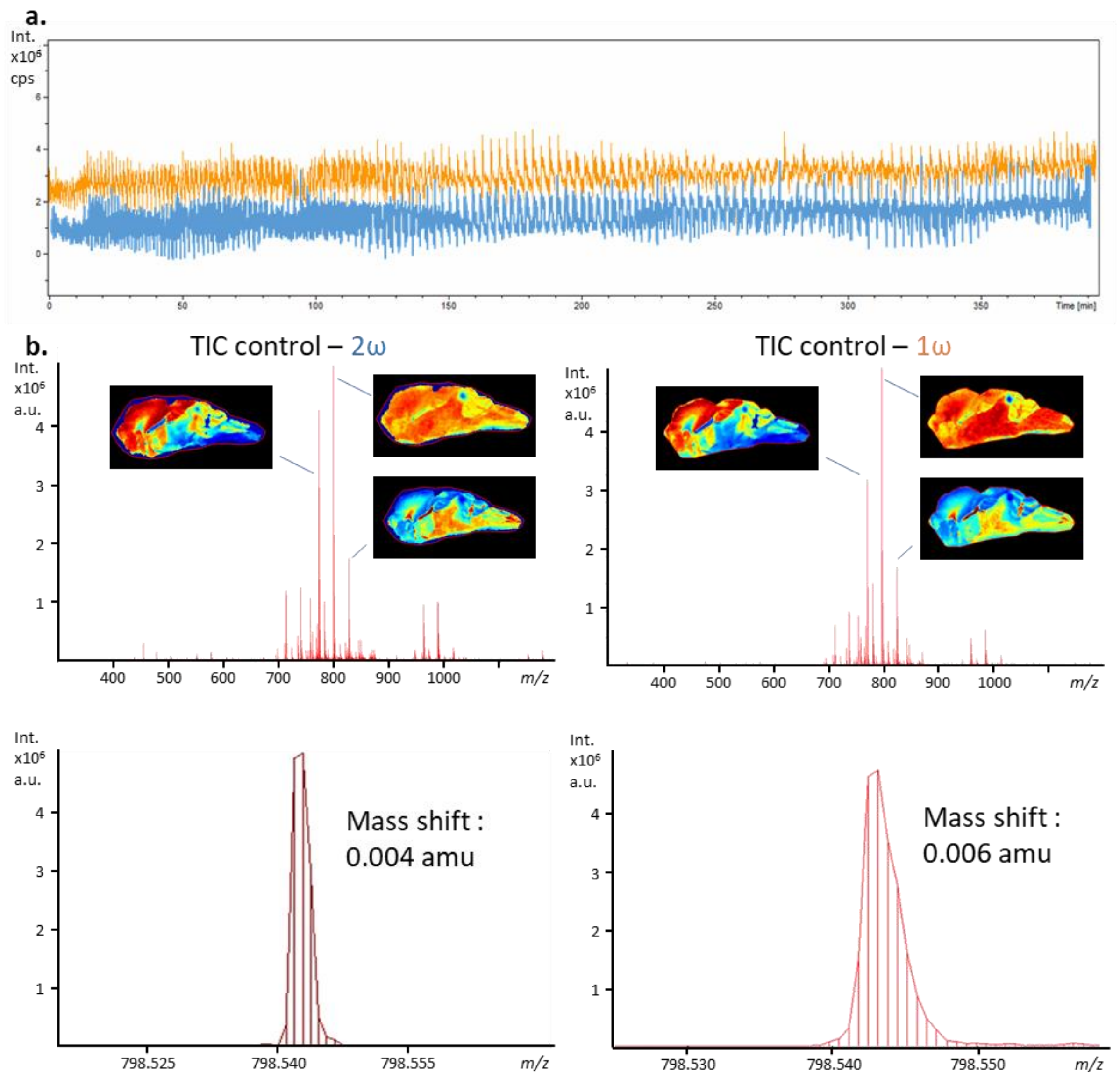

Figure S8. Total Ion Current over time for MSI acquisitions on a scimaX 2XR 7T in 1ω mode (orange) and 2ω mode (blue) (a). Centroided average mass spectra and distributions of *m/z* 772.53, 798.54 and 826.57 with a close-up on *m/z* 798.54 to compare the mass shift obtained with 1ω mode (right) and 2ω mode (left) (b).

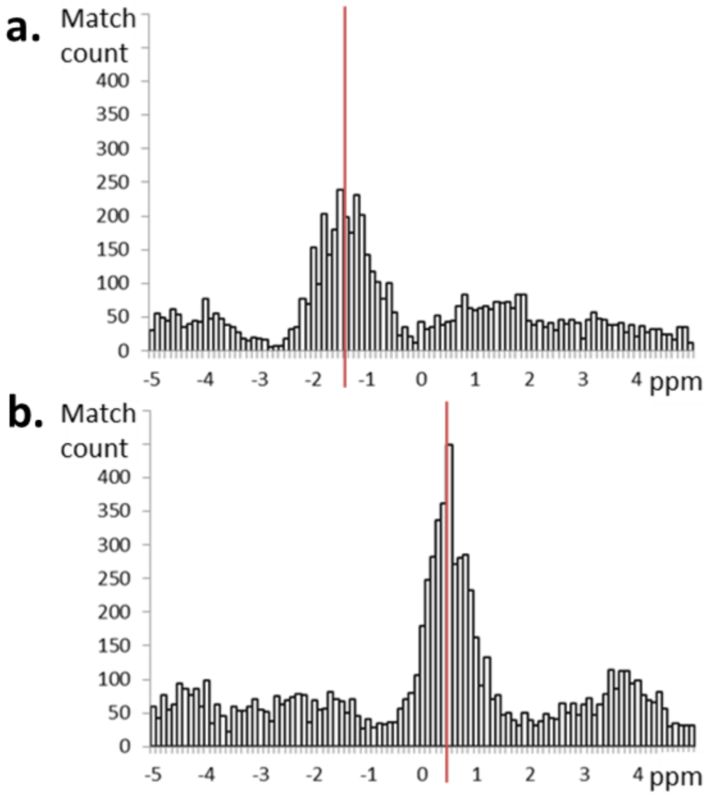

Figure S9. Representations of bulk searches using LIPID MAPS structure database (LMSD) submitting peak lists from MSI experiments. The number of matches versus their respective mass accuracy in ppm is presented. MALDI dynamically harmonized FT-ICR MSI datasets shown were acquired with unstable Total Ion Current (a) and stabilized Total Ion Current (b).

SUPPLEMENTARY TABLE

Table S1. Figures of merit of MSI images produced on 7T, 9.4T, and 21T MALDI FT-ICR instruments fitted with the dynamically harmonized cell.

**Articles**	Tiquet *et al.*		Bowman *et al.**
**MALDI MSI instrument**	Commercial dual ESI/MALDI source ScimaX 2XR 7T	Commercial dual ESI/MALDI source solariX XR 9.4T	Prototype hybrid linear ion trap – 21T FT-ICR w/ Paracell (modified Velos pro linear ion trap from Thermo)
**Processing mode**	Amplitude mode	Amplitude mode	Absorption mode
**Mass range**	Broadband *m/z* 300 - 1200	Broadband *m/z* 300 - 1200	N.D. (assumed to be narrowband m/z 700-900)
**Mass resolving power**	> 1,500,000 in single spec. @*m/z* 800 @16M @ 2ω<br>> 430,000 in average spec. @ 16M 1 or 2ω	> 400,000 in average spec. @ *m/z* 800 @ 4M<br>> 1,000,000 in average spec. @ *m/z* 800 @ 8M	> 1,600,000 @ m/z 400 (i.e. > 800,000 @ m/z 800)
**Mass calibration**	External	External	Internal
**Typical single spectra mass accuracy**	< 0.2 ppm @ 2ω	< 0.5ppm @ 1ω	< 0.1ppm
**Max. mass shift [a]**	±1.2ppm (±1.0 mDa) [c]	±0.6ppm (±0.5 mDa)	N.D. (assumed to be around ±0.4ppm)
**Dynamic range [b]**	50-100	50-100	500
**Transient duration**	Around 12 sec @ 16M using 2ω detection	Around 12 sec @ 8M using 1ω detection	Typical : 3.1sec
**AGC**	N.A.	N.A.	AGC disabled

Spec.: Spectrum

* Bowman and coworkers, Anal. Chem. (2020),92 :3133-3142; DOI : 10.1021/acs.analchem.9b04768
N.A: Not applicable

a. Maximum mass shift experimentally obtained from pixel-to-pixel *m/z* variation for the whole mass spectrometry image. FWHM in the average MSI data were considered to define the mass deviation in mDa.

b. Intra-scan dynamic range between the most abundant identified lipid and the least abundant identified lipids for the values reported by Tiquet *et al.* For Bowman *et al.*, the dynamic range value is the one claimed by the authors in the article.

c. Operating at extreme mass resolving power the scimaX 2XR 7T is more affected by limited TIC fluctuation due to the sample heterogeneity in regards to the solariX XR 9.4T and other FT-ICR using higher magnetic fields.

SUPPLEMENTARY GRAPH

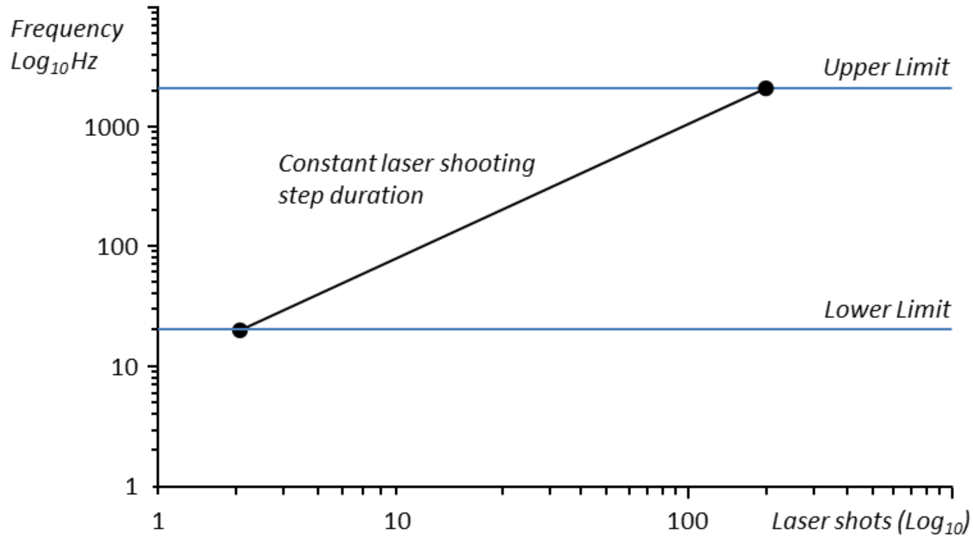

Graph S1. Logarithmic scale of the number of laser shots per second, i.e. frequency, versus the number of laser shots per MALDI ionization step, i.e. laser shots per scan. The laser frequency upper (2000Hz) and lower (20Hz) limits of the laser are indicated by blue lines and the effective ratio used to keep the laser-shooting time duration constant is shown by the black line. The slope corresponds to a ratio laser shots to laser frequency of 1:10.
